# The Human Throat as a Musical Instrument—(“Voice Culture.”)

**Published:** 1889-11

**Authors:** A. W. Calhoun

**Affiliations:** Prof. Diseases Eye, Ear and Throat, Atlanta (Ga.) Medical College


Vol. vi. NOVEMBER, 1889. No. 9.

### Original Communications.

### THE HUMAN THROAT AS A MUSICAL INSTRUMENT.—("VOICE CULTURE.")*

By A. W. CALHOUN, M. D.,

Prof. Diseases Eye, Ear and Throat, Atlanta (Ga.) Medical College.

Until a comparatively recent time there were profound mysteries difficult to explain, concerning the wonderful work of that part of the throat that we now know as the vocal cords. Investigators had long been occupied with researches into these mysterious depths, but until they had seen the larynx of a living being, it could only be proven that the voice was formed by the glottis, or mouth of the windpipe. For fifty years of the present century scientists had tried by mirrors and other appliances to examine the interior of the organ, but without result. Suddenly an inspiration came over Manual Garcia, the great singer and vocal teacher of London, whose observations and experiments, however, were made solely in the interests of vocal music. Ignorant of all trouble concerning the movements of the throat in the act of singing, he looked at himself. Using two mirrors, the

*Opening Address Atlanta Medical College.

one reflecting the image of the other, he saw the whole larynx clearly outlined. The publication of Garcia's discovery led the Austrian Prof. Türck, and the Hungarian Prof. Czermak, to employ the instrument for professional purposes; and in due time was evolved the laryngoscope, the revelations of which now teach us to thoroughly understand how words are produced and how the throat is able to send forth a wide variety of charming notes in singing.

The organ of voice, the larynx, is situated in man in the upper and forepart of the neck, where it forms a well known prominence in the middle line. It opens below into the trachea or windpipe, and above into the cavity of the pharynx, and it consists of a framework of cartilages connected by elastic membranes or ligaments, two of which constitute the true vocal cords. These cartilages are movable on each other by the action of various muscles, which thus regulate the position and the tension of the vocal cords. The trachea conveys the blast of air from the lungs during expiration, and the whole apparatus may be compared to an acoustical contrivance in which the lungs represent the wind chest, and the trachea the tube passing from the wind chest to the sounding body contained in the larynx.

If one looks down the larynx and trachea or windpipe of a singer as far as the bifurcation, (the point at which the windpipe separates into two tubes, one for each lung) a truly astonishing spectacle is presented. From the breast there rises to the middle of the neck, the passage of the air between the lungs and the mouth. At the lower end, it is divided into numerous branches —the bronchial tubes. At the upper end, like the capital of a column, is the larynx, resembling an angular box; strong cartilages make it very resistant, and the interior is lined with a mucous membrane, forming folds named the vocal lips (cords). These separate, lengthen or shorten in the formation of various sounds. The largest of the four cartilages rises in an angular form and protects the whole structure. It is only slightly shown in the neck of the woman, but is strongly marked in the man, and is properly called Adam's apple. Like everything else the larynx presents individual differences. A fine development is an indication of a powerful voice. As the child grows up there is a sudden alteration and increase of size, but it always remains smaller in the woman than in the man. The angles are less sharp in the woman, the muscles weaker, the cartilages thinner and more supple, which accounts for the sharp treble notes in the female voice.

It is interesting to watch the play of the organ by the aid of the laryngoscope, and see the changes which succeed one another in the low and high notes. At the moment when the sound issues, the glottis is exactly closed; then the orifice becomes a very long figure, pointed at the two extremities. As the sound rises the vocal lips approach each other and seem to divide the orifice into two parts; then as the highest notes are sounded, there is but a slit the width of a line. The vocal lips change like the glottis; they stretch out, broaden, thicken and vibrate more and more as the voice rises. Women, who have a smaller larynx and shorter vocal cords, can sing higher notes than men, with a tone less powerful but sweeter, more uniform and melodious.

In making comparisons to illustrate the articulated sounds of the throat, any one instrument would be entirely insufficient. Every musical instrument has some point of resemblance with the human voice, and yet, on the whole, all instruments are far inferior to this unique and wonderful contrivance in man, the human larynx.

An explanation of the production and inflection of the voice was a profound study to the early writers on this subject, and many curious comparisons were made.

*Ferrein* leaned to the notion that the vocal organ was like a string instrument. Dodart maintained that the larynx was a wind instrument. *Richeraud* and *Copeland* compared it to the French horn, and Cuvier to a flute. *Blumenbach* believed the larynx acted like an Æolian harp. *Magendie* and *Malgaigne* thought it was a reed instrument. Goeffrey St. Hilaire compared it to a reed and flute. Krattenstein had the prosaic idea that it was a drum. Savart thought it was similar to a bird call, and numerous other writers held that the action of the vocal cords was in some respect like the Jewsharp. Still farther back, the earliest writers were even more puzzled and their theories were numerous. Hippocrates, 400 B. C., says: "Man speaks by the air which he draws in the whole body, but especially in the cavities. Pushed to the exterior by the vacuum, the air engenders a sound, the tongue articulates by its clashings, intercepting it in the throat, and striking against the palate and the teeth, it makes the sound distinct."

Ninety years later, Aristotle defines the voice to be "a certain sound produced by a living body, for inanimate things have no voice." He compares the organs of the voice to a flute, the trachea being the body of the instrument.

Plato, 429 B. C., defines the voice to be "a rustling in the air reaching the soul through the ears."

Galen, 131 B. C., shows a far more intimate acquaintance with, the structure of the vocal apparatus, for he states the thorax to be the bellows or reservoir for air, the vocal cords the sonorous, vibrating body, by which the sound is produced, and the palate and mouth to correspond to the tube of a wind instrument, by which the sounds are modified, the whole apparatus being considered by him as analogous to a flute.

Claude Perrault, writing in the 17th century, thought he had solved the whole question when he said: "the voice is a sound of verberation, which the air enclosed in the chest excites in sallying forth violently, and in grazing the membranes constituting the glottis, so that it shakes its parts, and disseminates its particles, the return of which causes an agitation in the air capable of making an impression on the organ of hearing." He, too, classes the vocal organ with instruments of the flute kind.

To man alone belongs the full power of complete expression through the voice, and it is said, that every man possesses a peculiar tone of voice, equally as indicative of character as each feature of the face. Socrates divined the quality of a man's mind or soul by the tone of his voice. This great philosopher, on one occasion, beautifully and eloquently exclaimed; "speak, that I may see you." He makes such observations as these: "We perceive in a stutterer one that is easily enraged and as easily pacified, vain, officious, inconstant, and ordinarily quick."

"A coarse voice indicates a robust physique, a great talker, quick tempered, though conspicuously discreet."

"A piercing, fine or weak voice is indicitive of timidity, cunning, and generally of quick wit."

"An attractive and clear voice expresses a man who is prudent, sincere and ingenuous, but proud and incredulous; whereas, a firm voice, without harshness, denotes a person who is robust, intelligent, circumspect and benevolent."

"A full and sweet voice denotes a man who is peaceful, inclined to timidity, discreet and self-willed."

"A voice at first grave and then sharp and piercing means the quick temper of an impetuous, arrogant and impudent man; and he who possesses a trembling and hesitating voice is timid, weak, vain and sometimes jealous."

Musical tones are formed by the vibrations of the true vocal cords, and it is in the vocal tube, with its marvelous capacity for infinite combinations, that are formed all the qualities which the voice can possibly assume.

What ingenuity could devise an instrument, the cords of which might in a minute run through innumerable changes in contraction or relaxation, and through wonderful alterations in diameter and length; in an instant furnish different volumes of air, and give to it surprising variations in its force; imbed the instrument in materials by which its action was not impeded, but facilitated; lubricate the surface of the tube with a fluid that preserves its power, furnish it with an elastic appendage serving the purpose of bellows; and such auxiliary appendages which possess so great diversity in structure as to form density and component parts, and still all these dissimilar parts to be subservient to the volition and other functions? The human larynx is truly a wonderful instrument.

To form some conception of the variety and delicacy of the motions necessary to the adjustment of the vocal cords, listen to some singer whose voice commands with ease a great extent of the musical scale. For every single note there is a particular adjustment of the cords. In man it is calculated that the cords vary in length when in motion about one-fifth of an inch, and in woman one-eighth of an inch. Now the natural compass of most singers is about two octaves or twenty-four semitones. Between each semitone an ordinary singer can sound five or six distinct notes, so that one hundred and twenty would be only a moderate number of distinct sounds. He therefore produces one hundred and twenty different states of tension, and as the extreme variation of the cords is only one-fifth of an inch, the variation required to pass from one note to another is but the one-six hundredth of an inch.

A very expert singer can produce a much more delicate action than this. The celebrated Madame Mara, whose voice ranged through three octaves, could, it is said, produce between each tone—sound 100 different intervals, or 2100 in all, so that she could determine the contractions of her vocal ligaments to nearly 1-17000 of an inch.

Perhaps one of the most remarkable voices referred to in musical literature, having a range of three and a half octaves, is that of Farinelli, a famous eunuch singer. Many wonderful stories are told of the marvelous effects of his singing, both upon individuals and upon whole nations. He visited Madrid at a critical period in the life of Philip V of Spain, for that monarch had become such a prey to depression and melancholy that he totally neglected all the business affairs of his kingdom. When the great singer arrived, the queen arranged a concert at which the king could hear him without being seen. The effect was magical; the king seemed to take a new hold upon life, and Farinelli gained the respect, admiration and favor of the whole court. Philip considered that he owed his cure to the powers of Farinelli, and when the grateful monarch asked him to name his own reward, he answered that his best recompense was to know that the king had again become reconciled to performing the active duties of state. The final result was that the singer separated himself from the world of art forever, and accepted a yearly salary of 50,000 francs to sing for the king, as David harped for the mad king Saul. Every night, for the next ten years, he sang four songs for the king, without variation or change, and when Ferdinand VI, who was also a victim to his father's malady, succeeded to the throne, the singer continued to perform his minstrel cure, and acquired such enormous power and political influence that all court favor and office hung upon his breath. Farinelli amassed great wealth and continued his political power for many years, but no malicious pen has ever ascribed to him any of the corrupt arts by which royal favorites are wont to accumulate the spoils of office.

Man is not born a singer. Birds leave their nest with their voices rightly attuned and a repertory perfectly known. Man has to learn how to give his voice, and how to sing. Hence the culture of the voice; hence the art of singing. The graceful, noble, easy manners of a true aristocrat are the result of centuries of culture and refinement. They may, through study, be imitated by a clever actor, but he must never lose sight of his model. In the art of singing, one thing is certain—there is no short road to success. Unremitting study for months, even years, is the price to be paid. A study of the physiology of the voice is deemed unnecessary, as singing is an imitative art, and scientific knowledge does not improve the tone; but careful and systematic practice and devotion accomplish much, even where comparatively little to begin with was the starting point; but there must be good calibre of voice under all circumstances. Old Italian schools required their pupils to learn the art as they would a trade, spending from three to five years in technical studies, but we do not find such heroism in modern times. It is well to bear in mind in this connection, that the first knowledge of sounds and their signification to the brain can come *only* through the ear. There is no way to learn speech except through the organs of hearing; nor is there any other way for song. Porpora recognized the ear as the principal factor for voice production in the larynx. It is by the ear alone that we obtain the *harmony of the voices in voice.* There must exist a proper relation of ear and voice, and if this relation is seriously disturbed, all cultivation of the voice is fruitless.

In looking into the literature of this subject, I find the curious fact that nearly all the great musicians, and practically all the great singers, have risen up from the humble walks of life, and have been developed amid surroundings of poverty and in the stern struggle for existence. Nearly all the masters have been of lowly and obscure origin, and as a rule have lived and died in comparative poverty. The enduring music has been the child of poverty, the outcome of sorrow, and the apotheoses of suffering. Sebastian Bach was the son of a hireling musician. Beethoven's father was a dissipated singer. Cherubini came from the lowest and poorest ranks of life. Haydn's father was a wheelright, and his mother, previous to marriage, was a cook. Mozart's father was a musician in humble circumstances, and his grandfather a bookbinder. Rossini's father was a miserable, strolling horn-player, who led a wild Bohemian life. Schubert was the son of a poor schoolmaster, and his mother, like Haydn's, was in service as a cook at the time of her marriage. The divine Patti, the very queen of song, was born in Madrid of poor parents who taught music for a living. Schumann was a bookseller's son ; and Verdi the son of a Lombardian peasant. Wagner was born in humble circumstances, his father having been a petty municipal officer, and his step-father an unpretentious portrait painter, who at one time had also been a very poor actor.

Jenny Lind, the "Swedish nightingale," whose name shines among the very brightest in the golden book of singers, whose "every note was like a perfect pearl," and whom the Countess of Rossi (Henrietta Sontag) called "the first singer of the world," was born in the city of Stockholm, of poor struggling parents, who lived precariously by school-teaching.

Among all the great composers and singers but two were born in affluence—Meyerbeer and Mendelssohn; with these two exceptions, they developed the grandeur, the sublimity, the passion and the majesty of their music out of the storms of life, the pangs of sorrow and the hard battle with fate.

When the Babylonian tower shattered the universal language into innumerable dialects, singing remained the one expression of feeling common to all mankind. It is a language complete, and whoever understands this language thoroughly is capable of transferring the longings of his soul and the pearls of his thoughts into voice and ear of his hearers. It may be laid down as a fundamental and indisputable proposition, that music is the interpreter and the language of the emotions. It strikes every note in the gamut of human nature, from ecstatic joy to profound despair. It inspires, enrages, elevates, saddens, cheers and soothes the soul as no other one of the arts can. It gives voice to love, expression to passion, lends glory to every art, and performs its loftiest homage as the handmaid of religion.

